# AnimalMotionViz: An interactive software tool for tracking and visualizing animal motion patterns using computer vision

**DOI:** 10.3168/jdsc.2024-0706

**Published:** 2025-03-03

**Authors:** Angelo L. De Castro, Jin Wang, Jessica G. Bonney-King, Gota Morota, Emily K. Miller-Cushon, Haipeng Yu

**Affiliations:** 1 Department of Animal Sciences, University of Florida, Gainesville, FL 32611; 2 Laboratory of Biometry and Bioinformatics, Department of Agricultural and Environmental Biology, Graduate School of Agricultural and Life Sciences, The University of Tokyo, Bunkyo, Tokyo 113-8657, Japan

## Abstract

Monitoring the movement patterns of dairy cattle can provide important insight into space utilization or space occupancy in a barn. Although several precision livestock technologies have been developed to record dairy cattle movements, there is a lack of open-source tools to track and visualize group-level cattle movement patterns. Therefore, we developed an open-source computer vision software tool, AnimalMotionViz, that allows users to track and visualize group-level dairy cattle movement patterns using motion maps. The software comes with an easy-to-use web-based graphical user interface built with the Python Dash package. It implements a set of background subtraction algorithms in the OpenCV package to track animal motion patterns in real time. The software processes each frame of the input video and identifies the background and foreground using these algorithms. Foreground objects are then subtracted from the background across all frames and cumulatively overlaid on an empty mask image created with the first frame of the input video to visualize the intensity or frequency of motion across different regions. The user can generate a space-use distribution map in an image and video, a core and full-range map in an image, and also track specific regional motion with a custom mask. The software also returns the top 3 peak intensity locations, the total percentage of regions used, and the within-quadrant percentage of regions used. In four 5-min sample videos, quadrants with peak intensity of space use, as identified using the software, aligned with quadrants where calves spent the greatest duration of time, according to continuous recording of behavior from video. The space-use distribution and core and full-range maps generated by AnimalMotionViz can be used to understand space utilization or space occupation by dairy cattle, as well as to assess how space allocation affects their movement. Although AnimalMotionViz was developed to analyze dairy cattle data, its design provides the potential for broader application in studying the movement patterns of other animal species. We conclude that the newly developed AnimalMotionViz is a user-friendly and efficient tool to support research developments in precision livestock farming toward enhancing cattle management practices and improving pen designs.

## Highlights

• Monitoring dairy cattle movement patterns may reflect health and welfare.
• An interactive software tool was developed to monitor animal movement patterns.
• The software captures movement patterns using motion images, videos, and metrics.
• The software supports research on space use indicative of animal health and welfare.

## Graphical Abstract

**Figure:**
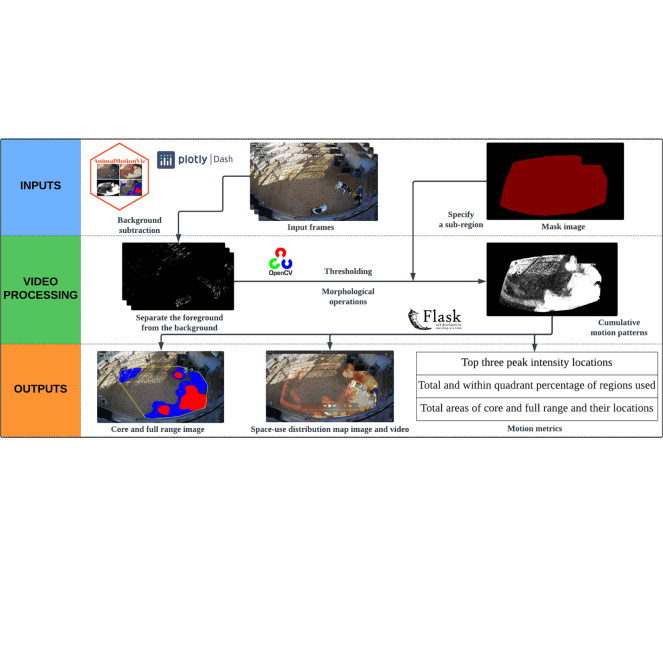
**Summary:** To provide novel insight into the movement and space use of dairy cattle, we developed AnimalMotionViz, an open-source software tool that processes video data to monitor animal movement patterns using computer vision. The software generates an image and video, a core and full-range image, and motion metrics that highlight and quantify movement intensity and patterns. This software tool is designed to support the broader adoption of computer vision systems to monitor changes in space use that are indicative of animal health and welfare.

The welfare and productivity of intensively housed dairy cattle is highly dependent on aspects of the housing environment (Bewley et al., 2017), and behavior is often interpreted as a key indicator of the welfare implications of housing management factors. For example, resting and feeding time are sensitive to stall (Krawczel et al., 2012) and feed bunk stocking density (DeVries et al., 2004), respectively. Although several previous studies have explored the impact of space allowance on dairy cattle (Schütz et al., 2015; Thompson et al., 2020; Ugarte Marin et al., 2024), quantifying how management practices influence the broader use of pen space remains challenging. To date, much research has focused on individual behavioral indicators using individual tracking data (Robert et al., 2009; Bailey et al., 2018; Alghamdi et al., 2022). Although these individual-level data can be aggregated and analyzed for group-level studies, such downstream analysis is complex and has limited the understanding of group-level movements or space use.

Improved understanding of dairy cow pen space utilization has potential applications related to both refinement of housing management and assessment of animal welfare. Specific use of space provided (e.g., preference for perimeter of the pen in calves; Morita et al., 1999; Ugarte Marin et al., 2024) may provide insight into preference or aversion for pen areas or specific features to refine decisions related to pen design. Similarly, information related to group-level or temporal variability in pen space use allows characterization of grouping or bunching behavior in dairy cows, which may be related to aspects of pen or barn design (van Schaik et al., 2021). Grouping behavior may additionally be subject to thermal conditions (Chopra et al., 2024), reflecting a potential group-level indicator of response to heat or cold stress. Space use is also influenced by individual factors, including social dominance (e.g., proximity to food sources; Nakanishi et al., 1992) and pain (e.g., increased use of a shelter in group-housed dairy calves following disbudding; Gingerich et al., 2020).

Tracking space use or occupancy may provide novel and useful information for improving livestock management, yet measurement of this data poses a challenge given limitations in visually monitoring all animals. Precision livestock farming uses a variety of sensor technologies and data-driven analytics to monitor or phenotype animals at the individual level (Morota et al., 2018). Wearable sensors have been widely used in livestock monitoring, but the high cost of applying them to large numbers of animals and the potential stress to the animals limit their practicality (Oczak et al., 2022). Computer vision, a subfield of precision livestock farming, analyzes images or video data from cameras and offers a noninvasive and cost-effective solution for monitoring livestock at both individual and group levels (Liu et al., 2020). Although several computer vision tools exist for tracking animal movements, they primarily focus on individual-level tracking and can be challenging to implement for group-level tracking. Thus, the use of computer vision to monitor group-level animal space utilization is limited in the literature due to the lack of publicly available and user-friendly software tools. Thus, our objective was to develop an interactive, open-source computer vision software tool for tracking and visualizing group-level animal motion patterns. Previous studies have shown that the use of an interactive graphical user interface (**GUI**) is useful to reach the user without specific training in data science (Morota et al., 2021) or computer vision (Wang et al., 2024). Here, we describe an interactive GUI based on Python Dash (Hossain et al., 2019), document the statistical methods and computer vision algorithms implemented in the software, and present examples that compare the output of the software with observations of space use and locomotor behavior.

AnimalMotionViz was developed entirely in Python, taking advantage of its versatility and ease of use to create a user-friendly software application. The Dash framework was used to build a GUI, chosen for its ability to streamline the creation of interactive and responsive software applications. The GUI of the AnimalMotionViz application is divided into input and output sections. The input section includes video processing parameters where the user can upload a video, select a color map, and adjust various settings. We replaced the file upload components in Dash, which do not support files larger than 200 MB, with the open-source dash-uploader (version 0.6.0; https://github.com/fohrloop/dash-uploader), which has no file size limitation other than the user's available disk space. The user input collected in this section is then passed to a callback function for processing and parsing, with the results returned to the GUI. The output section displays the results in 2 tabs and a summary table. The motion patterns image tab displays the final processed image, which represents the space-use distribution, with the top 3 peak intensity locations highlighted with symbols, as well as a core and full-range map image. The motion patterns video tab displays the processed space-use distribution map video using the Flask framework, version 3.0.3 (https://flask.palletsprojects.com/en/stable/), which is capable of efficiently streaming and serving large videos in the GUI. The summary table uses multiple metrics to quantify the motion patterns relevant to the motion maps. This structured layout ensures a clear and efficient presentation of both input parameters and output results, providing a comprehensive analysis of animal motion.

The development and implementation of AnimalMotionViz relies on several libraries and frameworks to ensure the efficiency of the application in producing the intended results. A user-friendly GUI is built with Dash, a Python framework for building interactive software web applications. The design and layout of the web application were refined with the Dash Bootstrap Components, and the Dash AG Grid was used to create data tables in the Dash application. The dash-uploader component is adapted for efficiently uploading large video files. The main processing engines are OpenCV (Bradski, 2000) for handling image and video processing and NumPy (Harris et al., 2020) for numerical computation in Python. The base64 module was used to encode images for uploading, decode the uploaded image data for downstream analysis, and re-encode the final motion pattern image for embedding in HTML for display in the Dash web application. The imageio library (version 2.36.0; https://github.com/imageio/imageio) converts the processed images into a video file, and the Flask framework is used to stream and serve the converted video for display within the Dash web application.

The first step is to upload a video of interest using supported file formats, including mp4, avi, mov, wmv, mkv, and flv. If the user is only interested in tracking a subregion of the video, they have the option of uploading a mask image created using annotation tools that allow a region of interest to be specified in the image, such as LabelMe (Russell et al., 2008) and Roboflow (Lin et al., 2022). Although this step is optional, it is recommended as it allows the definition of specific areas of interest to be considered during video processing, thereby increasing the focus and relevance of the analysis.

The uploaded video is converted into images (i.e., frames) and then undergoes background subtraction to detect animal movement by separating the foreground animals from the static background. The user can choose from several algorithms implemented with OpenCV, including Improved Mixture of Gaussians (**MOG2**), K-Nearest Neighbors (**KNN**), Geometric Multi-Grid (**GMG**), CouNT (**CNT**), Local Singular Value Decomposition (**SVD**) Binary Pattern (**LSBP**), and Google Summer of Code (**GSOC**). These algorithms are known for their varying strengths in handling various environmental conditions, such as lighting changes and background variability. The parameters of these algorithms were tuned using videos recorded under varying light conditions to determine and configure default values that ensure robustness to different lighting environments. The default background subtraction algorithm implemented in the software is MOG2, an adaptive algorithm that models each pixel independently as a mixture of Gaussian distributions over time to separate the foreground and background, allowing it to handle dynamic backgrounds and illumination changes (Zivkovic, 2004; Zivkovic and Van Der Heijden, 2006). The KNN algorithm is a nonparametric algorithm, which stores the history of pixel values for each pixel location and uses the k-nearest neighbors approach to classify the background (Barnich and Van Droogenbroeck, 2011). The third algorithm, GMG, combines statistical background image estimation with Bayesian segmentation. It requires an initial learning period and is sensitive to noise, but can achieve high accuracy once the learning phase is complete (Godbehere et al., 2012). The CNT algorithm is a real-time counter-based algorithm that tracks how often each pixel location remains unchanged to classify the background and foreground, making it efficient for handling illumination changes (Dey and Kundu, 2013). The LSBP algorithm uses local binary patterns combined with singular value decomposition to perform background subtraction, making it effective for handling background noise and dynamic changes (Guo et al., 2016). The GSOC was introduced to make LBSP faster and more robust.

Additionally, the user can specify the image processing interval to process every *n*th image (the default interval is 1). The software also allows the user to specify the kernel size and threshold value to perform a morphological operation on the detected motion to reduce small noise for generating the space-use distribution map and the core and full-range map, respectively. A small value removes small noise and preserves motion structure, while a larger value removes more noise but may also eliminate useful motion structure. The user can also adjust 2 weighting parameters and select a color map to visualize the detected cumulative motion patterns. The *α* and *β* weighting parameters are used to overlay the detected cumulative motion on top of the original frame to highlight the motion relative to the scene. This is achieved by combining the cumulative motion and the original frame with a weighted sum, where the *α* parameter is the weight of the original frame and *β* is the weight of the cumulative motion. To enhance the visualization of the cumulative motion, the user can select a color map from several color maps, including bone, ocean, pink, and hot, provided in the OpenCV library.

The software captures the uploaded video stored in local disk space and converts it to frames using OpenCV. If a mask file is provided, it is decoded from a base64-encoded string to binary data using the base64 module, and the decoded binary data are then converted to a NumPy array using the NumPy package for further analysis. The software then undergoes a user-selected background subtraction algorithm in OpenCV that distinguishes the foreground (i.e., moving animals) from the static background. Once the background is identified, the software processes frame by frame to detect motion and uses a binary image to represent the detected motion. The decoded and converted mask image (if available) is then applied to the motion image to restrict the motion detection to the specific region defined by the mask image using OpenCV. A morphological opening operation based on the user-specified kernel size parameter is then used to filter out small noise to obtain a cleaner and more accurate motion. The binary mask representing the detected motion in a given region is then accumulated over the frame. The centroid coordinates of the detected motion, filtered using a user-specified threshold value, are also accumulated over the frame.

The recorded cumulative motion data are first sorted by pixel intensity, and the coordinates of the 3 pixels with the highest intensity values, representing the most active locations, are retrieved. This cumulative motion data are then used to calculate the total and within-quadrant percentage of the region used. The total percentage of the region used is calculated as the total number of active pixels (i.e., pixel value >0) divided by the total number of pixels in the given view. For the quadrant-specific calculation, the view is divided into 4 equal sections (quadrants) by drawing a horizontal and a vertical line that intersect in the center of the view. Quadrants 1 through 4 correspond to the upper right, upper left, lower left, and lower right regions, respectively. The percentage of area used is then calculated for each quadrant using the same logic. The recorded cumulative centroid coordinates are processed using kernel density estimation to generate a density distribution of animal movements. From the generated density distributions, the core range and full range are calculated, representing high activity areas containing the top 50% and 95% of density values, respectively.

The recorded cumulative motion changes are first converted into a space-use distribution map image using a user-selected colormap and then blended with the original video frame via a weighted sum based on the specified weighting parameters, and saved to a temporary file. The retrieved top 3 peak intensity locations are marked on the blended space-use distribution map image with circle, square, and triangle symbols using OpenCV. The calculated core range and full range are visualized using a core and full-range map image, along with a convex hull drawn to highlight the outer boundary of the full range. The base64 module is used to encode the blended images into a base64 format for embedding and display in the motion patterns image tab in the software. A series of motion changes from the processed frames is converted into a space-use distribution map video using the imageio package, and the resulting video is stored as a temporary file on the local disk. The Flask framework is then used to stream and serve the video for display in the patterns video tab in the software, allowing users to play back and monitor the motion changes over time. In addition to these visualization outputs described above, a summary table is created and displayed in the Dash GUI using the Pandas package (The Pandas Development Team, 2020) to summarize the top 3 peak intensity locations, the total and within-quadrant percentage of regions used, and the total area of core range and full range in pixels, quantifying the spatial distribution and intensity of the animals' motion. The user has the option to download all these outputs locally for convenient access. The source code, detailed instructions, and video tutorials for AnimalMotionViz are available online at https://github.com/uf-aiaos/AnimalMotionViz.

To assess how output from the software aligned with space use and locomotor behavior, sample video files were selected for generation of space-use distribution maps and continuous observation of behavior. The sample video data used in this study featured Holstein heifer calves (28–42 d of age) housed in group pens (5 calves/pen) at the heifer rearing facility of the University of Florida Dairy Unit (Hague, FL). Four sample video clips of 5 min in duration were drawn from 24 h video recordings, collected using digital video cameras (Axis M2026-LE Network Camera, Axis Communications, Lund, Sweden) mounted in the center of the outside wall of the pen, ∼3 m from the ground, with video recorded at 15 frames per second to a network video recorder (Surveillance Station, Synology Inc., Bellevue, WA). These data were originally collected for a research trial where pens varied in effective space allowance (sections of the pen were blocked off to provide 3.7, 4.6, or 5.6 m^2^/calf) as described by Ugarte Marin et al. (2024), representing a range of common on-farm practices for raising dairy calves. For each video file, one observer (JBK) recorded location (within each quadrant), lying, stationary standing, and movement (walking or running) for each calf in the pen using Behavior Observation Research Interactive Software (BORIS; Friard and Gamba, 2016). These data were then summarized at the pen level as the average duration of each behavior (s/calf) by quadrant. Quadrants within each pen were then ranked based on total duration of time per quadrant and duration of time lying, standing, and moving per quadrant.

The GUI of the AnimalMotionViz application is shown in Figure 1. The software is divided into 2 main columns: the input section on the left and the output section on the right. The left column allows the user to upload a video and adjust various settings, including video processing parameters and color maps. The right column contains 2 tabs and a summary table. The motion patterns image tab displays the final space-use distribution map image with the top 3 peak intensity locations highlighted with colored circle, square, and triangle symbols, as well as the core and full-range map image. The patterns video tab displays the processed space-use distribution map video. The user can seamlessly toggle between the tabs. The summary table returns the motion metrics that quantify the motion changes over time. The first 3 rows detail the colors and symbols used to highlight the top 3 peak intensity locations, allowing for quick identification and interpretation of the most active areas in the video. These peak locations are important for pinpointing areas of substantial activity. The following 5 rows show the percentage of the region used in the entire view and within each of the 4 quadrants. The overall percentage quantifies how much space was occupied by animal movement, providing a baseline measure of overall activity levels. The quadrant-specific percentages are particularly useful for assessing the distribution of movement across different areas of the view, providing insight into spatial preferences or constraints in animal movement. By breaking down the analysis into quadrants, users can identify localized patterns of activity and compare the intensity of movement in different sections of the video. The last 2 rows show the total area of core range and full range in pixels, quantifying the overall space use in the pen.

**Figure 1:**
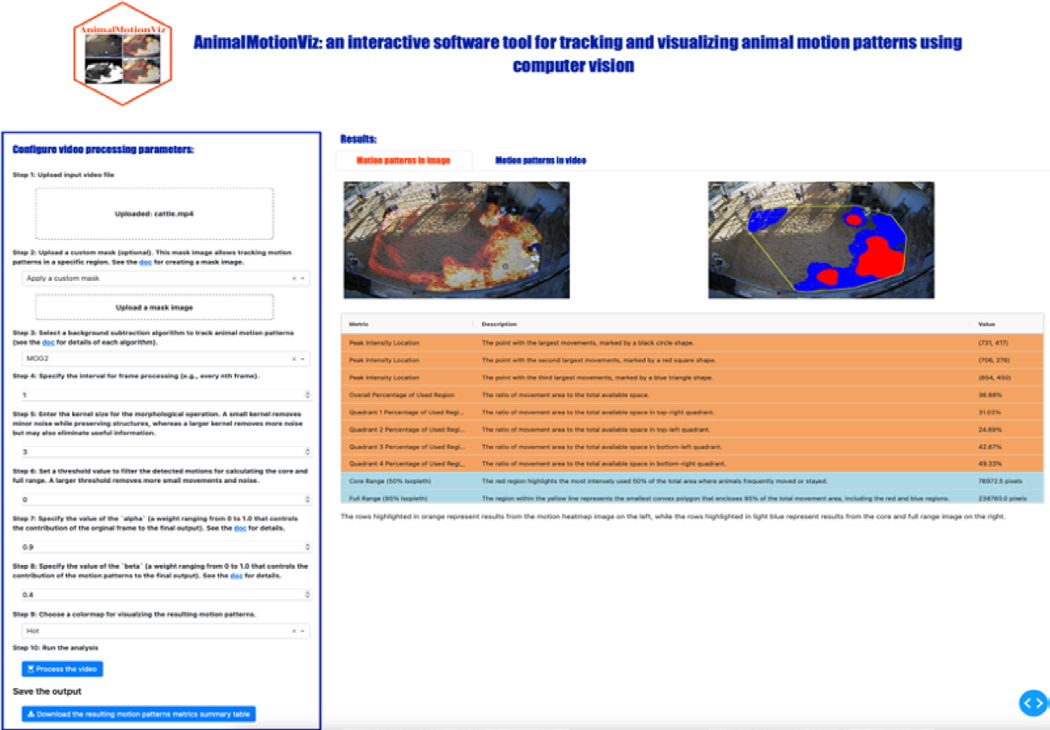
AnimalMotionViz graphical user interface. The left input panel allows the user to upload the input video and configure various parameters. The user also has the option to download the results. The right output panel displays the final space-use distribution map image and video, a core and full-range map, as well as a table with information on peak intensity locations and the percentage of the region used in the entire frame and in each quadrant.

The space-use distribution maps of 4 videos generated by the software are shown in Figure 2, and the rankings and durations of calf use and active movement observed during behavior analysis for each quadrant are summarized in Table 1. In Figure 2A, the top 3 peak intensity locations were identified in the upper left quadrant, which aligned with the quadrant with the greatest duration of calf use and active movement of walking or running, as indicated by the results for video 1 in Table 1. Figure 2B shows the top 3 peak intensity locations in the upper right quadrant, which was also consistent with the quadrant of the greatest duration of calf use and active movement based on video 2 observations in Table 1. In Figure 2C, the top 3 peak intensity locations were detected in the upper left quadrant, which corresponded to both the observed longest duration of calf use and active movement, compared with the durations in other quadrants, as shown in the results for video 3 in Table 1. Figure 2D shows the top 3 peak intensity locations in the upper left quadrant, consistent with both the observed longest duration of calf use and active movement for video 4 in Table 1.

**Figure 2:**
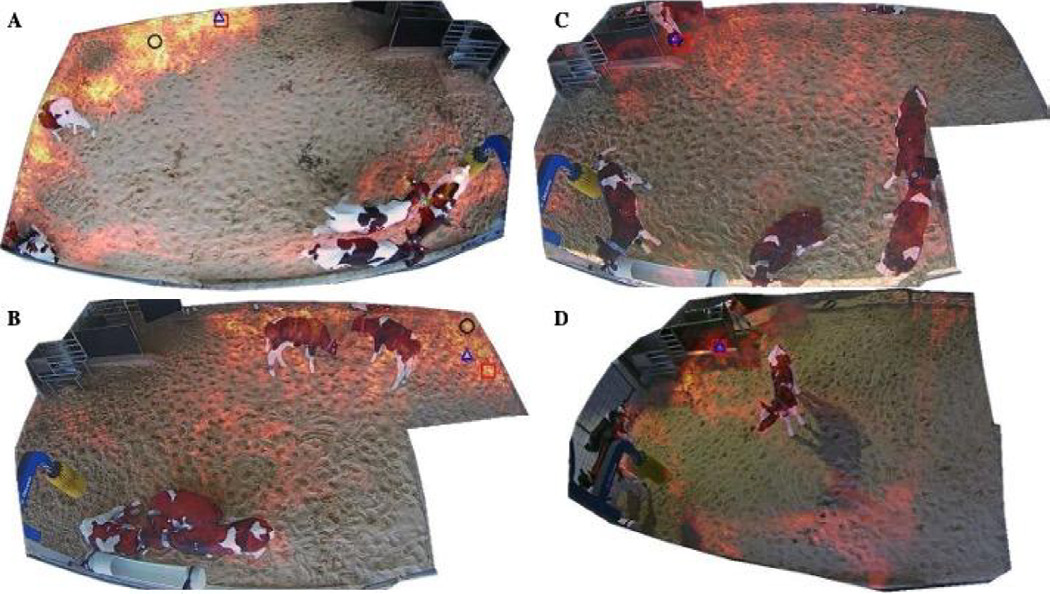
Space-use distribution maps tracking the use of pen space for 3 different group pens (A: 5.6 m^2^/calf, B and C: 4.6 m^2^/calf, and D: 3.7 m^2^/calf). Areas with more motion are shown in warmer colors (gold for the highest motion), and areas with less motion are represented in cooler colors (beige for no motion detected). The top 3 peak intensity locations are highlighted with black circles (first), red squares (second), and blue triangles (third).

**Table 1:** Rankings and durations of calf use and active movement within quadrants of group pens across 4 sample video clips

Video	Quadrant^1^	Calf use ranking and duration (s/calf)	Active movement ranking and duration (s/calf)
1	Upper left*	1 (103.4)	1 (21.7)
	Upper right	4 (3.8)	4 (2.1)
	Lower right	2 (102.4)	3 (6.7)
	Lower left	3 (40.7)	2 (8.0)
2	Upper left	4 (19.6)	4 (1.7)
	Upper right*	1 (188.6)	1 (45.9)
	Lower right	2 (39.6)	3 (5.8)
	Lower left	3 (2.3)	2 (9.5)
3	Upper left*	1 (162.6)	1 (28.8)
	Upper right	2 (49.6)	2 (21.4)
	Lower right	3 (23.8)	3 (5.7)
	Lower left	4 (14.9)	4 (2.7)
4	Upper left*	1 (115.3)	1 (11.8)
	Upper right	3 (15.2)	4 (1.3)
	Lower right	2 (114.6)	2 (7.5)
	Lower left	4 (3.9)	3 (0.8)

1 Quadrants marked with an asterisk indicate the quadrant with the top 3 peak intensity locations identified by the software, as shown in Figure 2, where space-use distribution map A, B, C, and D correspond to videos 1, 2, 3, and 4, respectively.

In the current study, an interactive tool, AnimalMotionViz, was developed to track and visualize animal motion patterns in images using computer vision. The flexible input column, which can be navigated by mouse clicks, is used to upload a video with no size limitation and to customize the motion pattern tracking parameters. The output column returns a space-use distribution map annotated image and video, along with a summary table that uses multiple metrics to quantify the motion patterns relevant to the map. The software can be used to understand pen space utilization and space occupation by dairy cattle in a barn. The output of the software can enhance management practices and lead to improved pen designs or housing conditions. It also provides an opportunity to study the relationship between space allocation and animal behavior in dairy cattle, with potential broader applications to other animal species. We believe the development of AnimalMotionViz will accelerate the broader adoption of computer vision systems to further support research developments in precision livestock farming.
